# On the packing density of the unbound protein-protein interaction interface and its implications in dynamics

**DOI:** 10.1186/1471-2105-16-S1-S7

**Published:** 2015-01-21

**Authors:** Jau-Ji Lin, Zih-Lin Lin, Jenn-Kang Hwang, Tsun-Tsao Huang

**Affiliations:** 1 Institute of Bioinformatics and Systems Biology, National Chiao Tung University, No.75, Bo-Ai St., East District, HsinChu City 300, Taiwan; 2 Institute of Biomedical Informatics, National Yang-Ming University, No. 155, Sec. 2, Linong St., Beitou District, Taipei City 112, Taiwan; 3 Bioinformatics Program, Taiwan International Graduate Program, Institute of Information Science, Academia Sinica, No. 128, Academia Rd., Sec. 2, Nankang District, Taipei City 115, Taiwan

## Abstract

**Background:**

Characterizing the interface residues will help shed light on protein-protein interactions, which are involved in many important biological processes. Many studies focus on characterizing sequence or structure features of protein interfaces, but there are few studies characterizing the dynamics of interfaces. Therefore, we would like to know whether there is any specific dynamics pattern in the protein-protein interaction interfaces. Thermal fluctuation is an important dynamical property for a residue, and could be quickly estimated by local packing density without large computation since studies have showen closely relationship between these two properties. Therefore, we divided surface of an unbound subunit (free protein subunits before they are involved in forming the protein complexes) into several separate regions, and compared their average thermal fluctuations of different regions in order to characterize the dynamics pattern in unbound protein-protein interaction interfaces.

**Results:**

We used weighted contact numbers (WCN), a parameter-free method to quantify packing density, to estimate the thermal fluctuations of residues in the interfaces. By analyzing the WCN distributions of interfaces in unbound subunits from 1394 non-homologous protein complexes, we show that the residues in the central regions of interfaces have higher packing density (i.e. more rigid); on the other hand, residues surrounding the central regions have smaller packing density (i.e. more flexible). The distinct distributions of packing density, suggesting distinct thermal fluctuation, reveals specific dynamics pattern in the interface of unbound protein subunits.

**Conclusions:**

We found general trend that the unbound protein-protein interaction interfaces consist of rigid residues in the central regions, which are surrounded by flexible residues. This finding suggests that the dynamics might be one of the important features for the formation of protein complexes.

## Background

Protein-protein interactions are involved in many biological processes such as signal transductions, enzymatic regulations, immunoglobulin recognitions, and allosteric controls [1-3]. A protein recognizes and interacts with its specific partner protein through a special surface region called interface [2,4-6]. It has been shown that the interfaces and non-interface regions vary in amino-acid composition [2,7-9], hydrophobicity [7,10], and types of secondary structures [8,11,12]. However, there are few studies characterizing the dynamical properties of interfaces. Generally, the thermal fluctuations of residues, one of the most important dynamical properties, could be inferred from the B-factors determined by X-ray crystallography or inferred from the order parameters determined by nuclear magnetic resonance (NMR) experiments. The routine experimental procedure usually examine the complete multi-protein assemblies, or protein complexes, therefore, the B-factors or the order parameters from these experiments only reflect the thermal fluctuations of residues in protein complexes. In order to obtain the thermal fluctuations of residues in unbound subunits, which are defined as free subunits without interacting with their partner subunits to form a complete complex, we use computational methods.

Molecular dynamics simulation [13-16] is a powerful method to compute the dynamical properties of proteins by integrating a long time trajectory; Normal Mode Analysis (NMA) [17-19] is another common choice to study the average protein dynamics. Nevertheless, these two methods are too computationally expensive to be used in the analysis of large datasets [20]. The Elastic Network Model and Gaussian Network Model (GNM) [21-23] provides a coarse-grained version of NMA to compute average dynamical properties of proteins. Recently, it has been shown [24] that the thermal fluctuations of a residue can be estimated by some structure-derived properties such as contact number (CN model), distance to protein fixed-point (PFP model), and weighted contact number (WCN model). The CN model showed that the B-factor is related to the number of noncovalent neighboring atoms within a cut-off distance [25]. The PFP model demonstrated that the atoms lying on the same shell centered at the fixed point of the protein tend to have similar B-factors [20]. The WCN model reported that the B-factors are quantitatively correlated with its weighted contact number, where the weight being the square of the reciprocal distance between the contacting pair [26]. Comparing these structure-derived B-factor profiles with the experimental X-ray B-factors, the WCN model predicts better B-factors than CN model, PFP model and GNM [26]. Take triosephosphate isomerase (a homodimeric enzyme, PDB code: 1YPI) as an example, it is shown that the residues with larger B-factors (i.e. more flexible, colored red in Fig. S1A, Additional file 1) have smaller WCN (colored red in Fig. S1B), and vice versa. Figure S2 (Additional file 1) compares the normalized B-factor profiles with the normalized reciprocal WCN profiles, showing close association between B-factor and WCN.

Since weighted contact number (WCN) provides a simple, fast means to estimate the B-factors of residues, we used it to quantify thermal fluctuations for the residues in the unbount subunits. We analyzed the WCN distributions of interface residues in unbound subunits collected from a dataset containing 32 non-homologous homodimers [12], and then we analyzed four large datasets of protein complexes that respectively consist of 793 homodimers, 274 heterodimers, 115 obligate complexes, and 212 transient complexes. The homodimers and heterodimers were collected from 3D complex [27]; the obligate complexes and transient complexes were collected by Weng's group [28]. Our results showed that the interfaces of the unbound subunits from various complex datasets share same preference: the residues in the central regions (defined as residues thoroughly buried upon complex formation) have larger WCN than non-interface residues, while the residues in the peripheral regions (defined as residues partial exposed to solvent upon complex formation) have smaller WCN than non-interface residues. For WCN are negatively related to the thermal fluctuations [25,26], our results reveal the specific dynamics pattern in the unbound protein-protein interaction interfaces: the central regions consist of rigid residues, while the peripheral regions consist of flexible residues. This finding suggests that protein dynamics might be an important factor for protein-protein interaction.

## Methods

### The weighted contact number (WCN)

The weighted contact number of the *i*th residue is the summation of the square of reciprocal distance between the other neighbor residues in the same chain [26]:

$$w_{i}=\overset{N}{\underset{j\neq i}{\sum }}\frac{1}{r_{ij}^{2}}$$

**Figure 1:**
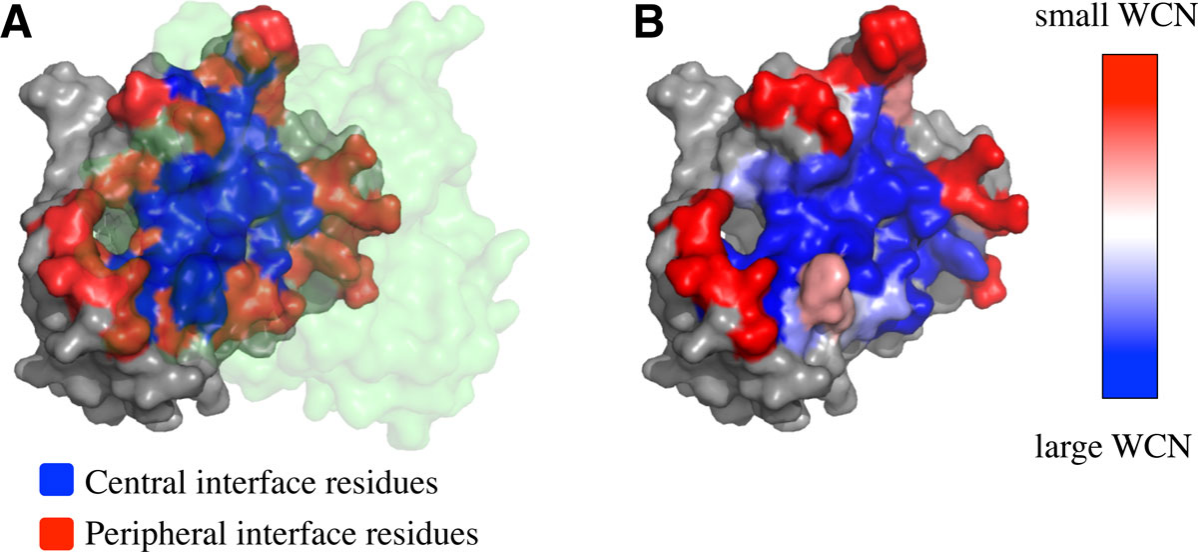
**Example of surface subregions and the degree of packing density**. The interface of the thymidylate synthase (PDB code: 2TSC). (A) One of the two identical subunits interacts with its partner (translucently green region) through the interface. The central interface region is colored red, the peripheral interface region is colored red, and the non-interface region is colored gray. (B) The colors of interface residues are ramped from blue (large WCN, i.e., high packing density, more rigid) to red (small WCN, i.e., small packing density, more flexible), and the non-interface residues are shown in gray.

The reciprocal value of WCN has been shown closely related to the thermal fluctuation of a residue [25,26], so we could estimate the thermal fluctuations of residues from their WCN. In order to obtain the thermal fluctuations of residues in the unbound subunits, only the residues in the same subunit were considered as neighbor residues when we evaluated the WCN of each residue. Since the partner subunits in a complex were not used in the calculation, the WCN would only reflect the thermal fluctuations of residues in unbound subunits. Figure 1B shows the WCN of residues in an unbound subunit from a thymidylate synthase.

It should be noticed that based on the equation, the size of protein has influence on WCN. In order to compare WCN among protein subunits with various sizes, we normalized the WCN to its corresponding z-scores: $z=\frac{w-\bar{w}}{\sigma_{w}}$ for each protein subunit, where $\;\bar{w}$ and *σ_w _*were the mean and standard deviation of the WCN based on all the surface residues in an unbound subunit. By the above procedures, and the average normalized WCN was denoted as 〈*z*〉. For a global analysis, WCN were not normalized for each individual subunits but normalized for the whole residues in a dataset, and this normalized WCN was denoted by *zWCN*. Therefore, the average normalized WCN was denoted as 〈*zWCN*〉 for a global analysis. In summary, high WCN residues are more rigid while low WCN residues are more flexible.

### Definition of protein-protein interfaces and the subregions of interface

The surface residues of a protein subunit were those residues whose solvent accessible surface area is not zero in the unbound subunit, where the solvent accessible surface areas of residues were calculated by the program dsspcmbi [29] with a water molecule radius of 1.4 Å. After that, a surface residue was considered in interface if any of its heavy atoms contact with any other partner proteins within 5 Å [30-32]. The interface residues were considered in the central region of interfaces if their relative solvent accessible surface areas in a complex were smaller than or equal to 5%, and were called cnetral interface residues; the rest of interface residues were considered as peripheral interface residues. The relative solvent accessible surface area of a residue was equal to its real accessibility dividing by maximal accessibility [33]. Figure 1A illustrates the central interface residues (blue region) and peripheral interface residues (red region) in the unbound subunit, while the non-interface residues are drawn grey. The surfaces and interfaces are drawn with PyMOL.

To compare the difference of WCN distributions among different regions on the surface of unbound subunits, we compared their average normalized WCNs, denoted by 〈*Z*〉_*N*_, 〈*Z*〉_*C*_, and 〈*Z*〉_*P*_, where N, C, and P stand for non-interface, central interface and peripheral interface, respectively. If the normalization is global, the average normalized WCN were denoted by 〈*zWCN*〉_*N*_, 〈*zWCN*〉_*C*_, and 〈*zWCN*〉_*P*_.

### Datasets of protein-protein interactions

We used the 32 non-homologous (sequence identity of <35% and structur-ally different) protein homodimers [12] to show the WCN distributions of residues in different surface subregions. In this dataset, all structures are determined by X-ray crystallography except interleukin 8 (PDB code: 1IL8). For this NMR structure, we used the first model for our analysis. Three PDB structures 2SDH, 2SSI, 3GAP were obsolete and were substituted with 3SDH, 3SSI, 1G6N respectively. Table S1 shows the list of protein-protein interaction pairs in this homodimer data set.

In addiction, we compared the WCN distributions in homodimers with the distributions in heterodimers. There are 793 non-redundant homodimers (listed in Table S2) and 274 heterodimers (listed in Table S3) selected from 3Dcomplex [27] database with sequence identity threshold of 20%. In order to compare the WCN distributions of different surface regions by student *t*-test, the unbound subunits that have only one or zero central interface residue were removed.

We also applied WCN on obligate and transient complexes. The non-redundant structural dataset manually separated into 115 obligate complexes and 212 transient complexes [28], whose sequence identity are smaller than 25%. The unbound subunits that have less than one central interface residues were removed because the WCN distributions were compared by student *t*-test. Table S4 and S5 respectively list the unbound subunits from obligate and transient complexes.

## Results and discussion

### Characteristics of datasets

There are five protein complex datasets used in this study and are summarized in Table S6. For the residues in these various datasets, 87% to 89% residues are on the protein surfaces; among these surface residues, 12% to 20% are in the interfaces. The percentages of interface residues on the surfaces are similar for homodimers (18.4%) and heterodimers (19.8%), but are slightly different between obligate complexes (15.7%) and transient complexes (12.4%). Considering the interface residues, about one-third of the residues were in the central regions. The proportions of residues in the central regions of interfaces are similar for homodimers (31.7%) and heterodimers (32.5%), but are different between obligate and transient complexes (36.1% and 30.7%, respectively). Our analysis suggests that the interfaces in obligate complexes tend to be larger than interfaces in transient complexes. Moreover, the central interface regions in obligate complexes tend to be larger than those in transient complexes if their interface sizes are the same.

### WCN distributions of residues from different surface subregions

**Figure 2:**
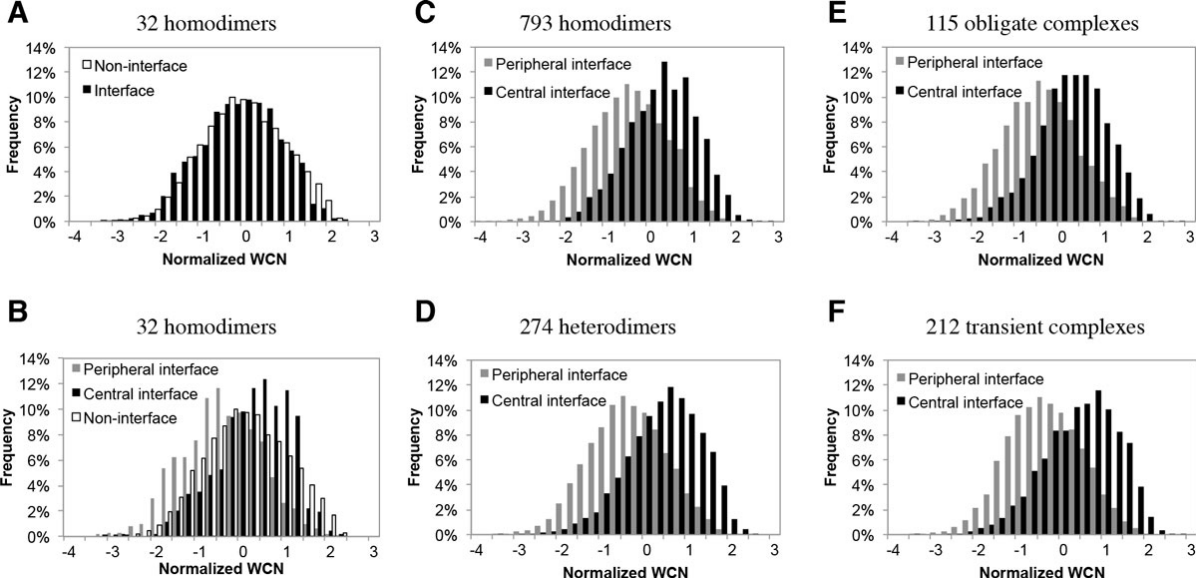
**Distributions of normalized WCN in various surface subregions**. The WCN distributions in various surface subregions from datasets composed of **(A) **and **(B) **32 homodimers, **(C) **793 homodimers, **(D) **274 heterodimers, **(E) **115 obligate complexes, and **(F) **212 transient complexes.

Figure 1A shows the difference surface subregions of thymidylate synthase (PDB code: 2TSC): central interface region is colored blue and peripheral interface region is colored red. The color of Figure 1B is based on the value of WCN, showing that the center interface residues have larger packing densities (more rigid) while the peripheral interface residues have smaller packing densities (more flexible). In order to know whether the interfaces vary in packing density for different types of homodimers, we analyzed the interfaces from 32 non-redundant homodimers [12]. Though there is no clear difference between the interface and the non-interface residues in Figure 2A, it appears significant distinction when the interfaces are further divided into central regions and peripheral regions shown in Figure 2B, where the normalized WCN distribution of central interface residues (*zWCN_C_*, black bars) tend to be larger than that of non-interface residues (*zWCN_N_*, white bars) as well as the WCN distribution of peripheral interface residues (*zWCN_P_*, grey bars) tend to be smaller than non-interface residues.

We performed the Student's *t*-test to evaluate the differences among these normalized WCN distributions, denoted by *zWCN*, by MATLAB. Both the *P*-values of *zWCN_C _*- *zWCN_N _*and *zWCN_P _*- *zWCN_N _*are smaller than 3.17 × 10^-12^, and the *P*-value of *zWCN_C _*- *zWCN_P _*is even as small as 7.83 × 10^-83^, indicating that the central interface residues have significantly larger WCN than non-interface residues, and the peripheral residues have significantly smaller WCN than non-interface residues.

Besides the average WCN values of residues for the whole dataset, we calculated the average WCN values of residues in different surface subregions of each individual unbound subunit, denoted by 〈*z*〉_*C*_, 〈*z*〉_*P *_and 〈*z*〉_*N*_, where C, P and N represented central, peripheral, and non-interface regions. We found that 〈*z*〉_*C *_is larger than 〈*z*〉_*P *_in all 64 unbound subunits from the 32 homodimers (Fig. S3A, Table S1). When compared with 〈*z*〉_*N*_, 〈*z*〉_*P *_is smaller than 〈*z*〉_*N *_in 91% interfaces (58/64), and 〈*z*〉_*C *_is larger than 〈*z*〉_*N *_in 75% (48/64) interfaces (Fig. S3B, Table S1). These results showed that the relationship between the average WCNs from different surface subregions holds not only for all the residues across the datasets, but also for the residues in every unbound subunit.

**Figure 3:**
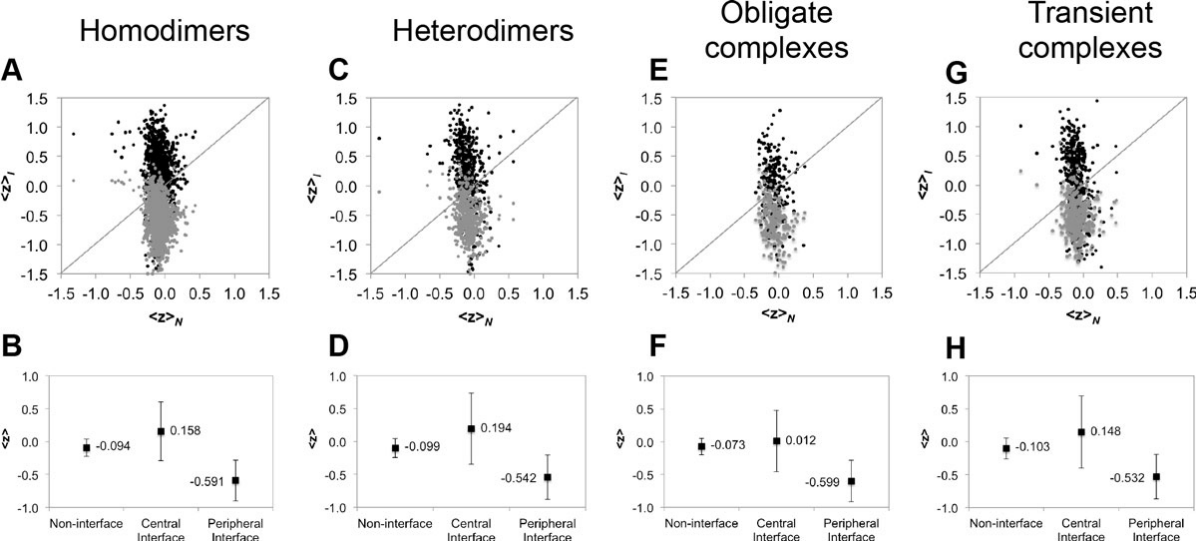
**Comparison of the average WCN in subregions of various datasets**. Comparison of average WCN values among different surface subregions from datasets composed of **(A) **and **(B) **793 homodimers, **(C) **and **(D) **274 heterodimers, **(E) **and **(F) **115 obligate complexes, and **(G) **and **(H) **212 transient complexes. Each point represents a pair of the average WCN values in different surface subregions from a unbound subunit. A black point represents 〈*z*〉_*C*_, 〈*z*〉_*N*_), and a grey point represents 〈*z*〉_*P*_, 〈*z*〉_*N*_), where 〈*z*_*C*_〉, 〈*z*〉_*P*_, and 〈*z*〉_*N *_represent the average WCN values of central interface, peripheral interface, and non-interface residues respectively. The figures in the second row present the means and standard deviations of 〈*z*〉 from the respective datasets.

In addiction, we analyzed a larger dataset comprising 793 non-homologous homodimers collected from 3D complex [27]. The WCN distributions of central and peripheral interface residues (Figure 2C) are similar as those in the 32 homodimers dataset (Figure 2B). The relationship among central interface residues, non-interface residues and peripheral interface residues can be expressed as the inquality: 〈*zWCN*〉_*C *_> 〈*zWCN*〉_*N *_> 〈*zWCN*〉_*P*_, where 〈*zWCN*〉_*Y *_denotes the average normalized WCN in the surface subregion Y (Table 1 and Figure 3B). Our results suggested that a homodimer interface are composed of residues with larger WCN surrounded by residues with smaller WCN, and the average packing density of non-interface residues are between those of the central and peripheral interface residues.

**Table 1:** The average WCN in surface subregions

Dataset	〈*zWCN*〉_*N*_	〈*zWCN*〉_*C*_	〈*zWCN*〉_*P*_	〈〈*z*〉_*N*_〉	〈〈*z*〉_*C*_〉	〈〈*z*〉_*P*_〉
793 homodimers	-0.103	0.146	-0.598	-0.094	0.158	-0.591
274 heterodimers	-0.111	0.247	-0.553	-0.099	0.194	-0.542
115 obligate complexes	-0.098	0.070	-0.625	-0.073	0.012	-0.599
212 transient complexes	-0.118	0.256	-0.532	-0.103	0.148	-0.532

N, C, and P denote non-interfaces, central interfaces and peripheral interfaces. 〈*zWCN*〉_*X *_is the average value of normalized WCN collected from all residues in subregion × in a data set. 〈〈*z*〉_*X*_〉 is the mean of 〈*z*〉_*X*_, which is the average value of normalized WCN collected from residues in subregion × in an unbound subunit.

### The WCN distributions of homodimeric interfaces and heterodimeric interfaces

We used the 274 heterodimers dataset collected from 3D complex [27] to compare the WCN distributions of interface residues between homo- and heterodimers. The distributions of different surface subregions from heterodimers are shown in Figure 2D. As we can see from the figures, these distributions are quite similar between homo- and heterodimers. Figure 3C and 3D show that central interface residues tend to have larger WCN than peripheral interface residues, the same as the cases from homodimers. For the homodimers dataset, the average value of WCN of central interface residues 〈*zWCN*〉_*C *_= 0.146 is significantly larger than that of peripheral interface residues 〈*zWCN*〉_*P *_= -0.598 (Table 1, first row column 2 and 3); for the heterodimers dataset, the average value of WCN of central interface residues 〈*zWCN*〉_*C *_= 0.247 is also significantly larger than that of peripheral interface residues 〈*zWCN*〉_*P *_= -0.553 (Table 1, second row column 2 and 3). As the cases for homodimers and heterdimers, the average values of WCN of non-interface residues are between the average values of central and peripheral interface residues.

The above result is from global analysis. When comparing packing density distribution subunit-by-subunit, 98.4% unbound interfaces (1435/1459) from the 793 homodimers dataset obeyed the inequality 〈*z*〉_*C *_> 〈*z*〉_*P *_, where 〈*z*〉_*C *_and 〈*z*〉_*P *_were the average values of central and peripheral interface residues in a unbound subunit (Figure 3A, Table S2); moreover, 94.2% unbound interfaces (467/496) from the 274 heterodimers dataset also obeyed the same inequality (Figure 3C, Table S3). These results indicated that both homo- and heterodimer interfaces were composed of residues with larger WCN in the central regions surrounded by residues with smaller WCN in the peripheral regions (Figure 3B and 3D).

Comparing homodimeric interfaces with heterodimerics interfaces, the former have smaller 〈*zWCN*〉_*C *_than the later (see the second column in Table 1). These results implied that the central residues in homodimers have less neighbors than those in heterodimers. As the complex are usually symmertric for homodimers, the interface residues are in the center of the complexes. Hence, the interfaces are usually more flat than other surface regions, so the neighbor residues are only in half of the space divided by the interface plane. In contrast, a heterodimer usually consist of one large subunit with concave interface and one small subunit with convex interface. Thus, the number of neighbors for a central residue in concave interface would be more than that in flat interface, and this causes the higher WCN in the large subunits. Although with the same deduction the WCN is smaller in the small subunits, the number of central residues in a large subunit is more than that in a small subunit. Therefore, the finally 〈*zWCN*〉_*C *_of heterodimers is larger than 〈*zWCN*〉_*C *_of homodimers.

### The WCN distributions of interfaces in obligate complexes and transient complexes

Both the 115 obligate complexes and 212 transient complexes collected by Mintseris et al. [28] have the same trend in WCN distribution as the dimers (Figure 2E and 2F): their central interface residues tended to have larger WCN than peripheral residues (details are in Table S4 and S5). Analyzing each interface of unbound protein subunits from 115 obligate complexes, 96.4% unbound interfaces (215/223) obeyed the inequality 〈*z*〉_*C *_> 〈*z*〉_*P *_(Figure 3E, Table S4); for 212 transient complexes, 90.5% unbound interfaces (361/399 interfaces) obeyed this inequality (Figure 3G, Table S5). These results indicated that both obligate and transient interfaces are also composed of residues with larger WCN in the central regions surrounded by residues with smaller WCN in the peripheral regions (Figure 3F and 3H).

Comparing obligate interaction with transient, the obligate complexes significantly differed from transient complexes in 〈*zWCN*〉_*C *_(the values were 0.070 and 0.256 respectively, in Table 1), implying that the central regions of the unbound interfaces in transient complexes are more rigid than those in obligate complexes. This phenomenon could be explained by the reason that many transient interfaces were composed of catalytic sites, and for an enzyme/ligand complexes, which are transient complexes, and previous studies have shown that the active sites are proximal to the protein centroid [34] so that the active sites are expected to be more rigid [35].

## Conclusions

For the interface subregions in unbound protein subunits, our analyses showed the distinct distributions of packing density, suggesting distinct thermal fluctuation. These distinct thermal fluctuations reveal specific dynamics pattern in unbound interface: interface regions consist of rigid residues in the central regions, and the peripheral regions consist of flexible residues. This specific dynamical feature appear in various protein complexes including homodimers, heterodimers, obligate complexes, and transient complexes, based on the average packing density of surface subregions with 〈*zWCN*〉_*C *_> 〈*zWCN*〉_*N *_> 〈*zWCN*〉_*P *_(central interface regions, non-interfaces, peripheral interface regions).

Previous studies have divided an interface into buried region (core region) and exposed region (rim region) upon complexation, and have found that this two regions vary in sequential, structural, and evolutionary features such as amino acid composition, degree of sequence entropy and sequence conservation [1,5,7,36], and secondary structure propensity [37]. Our results also present that the unbound interfaces have distinct distributions of packing density when dividing an interface into buried region (central regions) and exposed region (peripheral region). Sine previoius studies [25,26] have shown the structure-dynamics relationship between the packing density and thermal fluctuation, our finding implied specific dynamics pattern in the interfaces of unbound protein subunits. For characterizing a specific interface by a combination of interface parameters is suitable [4], combining the specific dynamical feature with sequential and structural features might help us to more understanding in protein-protein interaction.

The mechanism for protein-protein association was reviewed that a temporary complex is formed through nonspecific collisions guided mostly by electrostatic interactions, and then this temporary complex reorientates to form its final structure guided mostly by desolvation [38]. The rate of this association is modulated by the perturbations in charge distribution [39], and our results suggested that these perturbations may arise from the flexibility of the charged or polar residues in the peripheral interfaces. On the other hand, it has been shown that hydrophobicity stabilizes protein-protein interactions, and geometric and electrostatic complementarity plays a selective role in deciding which proteins could interact with each other [5,40], and our results suggested that these specific interactions may arise from the rigidity of the residues in the central interfaces. In conclusion, our findings implied that the flexibility of peripheral interface residues facilitates a protein to capture the candidates of its interaction partners by the perturbations in charge distribution, and then the rigidity of central interface residues helps the protein to select the exact partner by structural and physicochemical complementary. Finally, this interaction is stabilized by hydrophobic effect. However, resolving these issues is beyond the scope of the present report. To sum up, our results suggested that the dynamics might be an important feature for the formation of protein complexes.

## Competing interests

The authors declare that they have no competing interests.

## Authors' contributions

JJL analyzed data, performed the statistical analysis.

ZLL and TTH collected the datasets, wrote the codes and performed the calculations.

JKH and TTH conceived and designed this study.

JJL and TTH drafted the manuscript.

## Supplementary Material

Additional file 1 Figure S1 - A structure of triosephosphate isomerase colored by B-factor and by WCN. Figure S2 - Comparison of the B-factor and WCN profiles of triosephosphate isomerase. Figure S3 - Comparison of the normalized WCN in difference surface subregions for the small homodimer dataset. Table S1 - The list of the protein-protein interaction pairs from 32 homodimers.Table S2 - The list of the protein-protein interaction pairs from 793 homodimers. Table S3 - The list of the protein-protein interaction pairs from 274 heterodimers. Table S4 - The list of the protein-protein interaction pairs from 115 obligate complexes. Table S5 - The list of the protein-protein interaction pairs from 212 transient comoplexes. Table S6 - The protein complexe datasets analyzed in this study. Click here for file
