# Supplementary figures and images for: On the packing density of the unbound protein-protein interaction interface and its implications in dynamics
Source: BMC Bioinformatics. 2015 Jan 21;16(Suppl 1):S7. doi: 10.1186/1471-2105-16-S1-S7 (PMC4331706; doi:10.1186/1471-2105-16-S1-S7)

**A**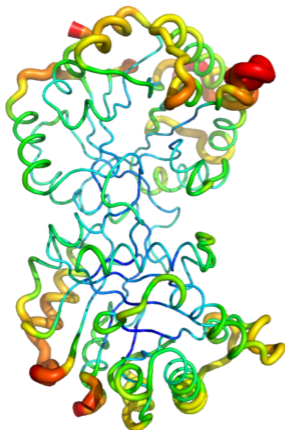

large B-factor

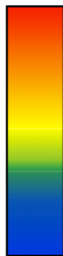

small B-factor

**B**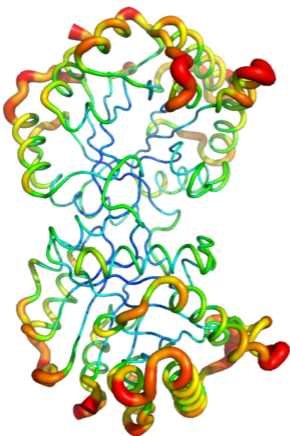

small WCN

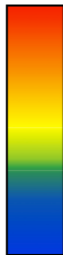

large WCN

Supplement: Additional file 1 — Figure S1 - A structure of triosephosphate isomerase colored by B-factor and by WCN. Figure S2 - Comparison of the B-factor and WCN profiles of triosephosphate isomerase. Figure S3 - Comparison of the normalized WCN in difference surface subregions for the small homodimer dataset. Table S1 - The list of the protein-protein interaction pairs from 32 homodimers.Table S2 - The list of the protein-protein interaction pairs from 793 homodimers. Table S3 - The list of the protein-protein interaction pairs from 274 heterodimers. Table S4 - The list of the protein-protein interaction pairs from 115 obligate complexes. Table S5 - The list of the protein-protein interaction pairs from 212 transient comoplexes. Table S6 - The protein complexe datasets analyzed in this study. [file 1471-2105-16-S1-S7-S1.pdf]
